# Limb patterning genes and heterochronic development of the emu wing bud

**DOI:** 10.1186/s13227-016-0063-5

**Published:** 2016-12-20

**Authors:** Craig A. Smith, Peter G. Farlie, Nadia M. Davidson, Kelly N. Roeszler, Claire Hirst, Alicia Oshlack, David M. Lambert

**Affiliations:** 1 Department of Anatomy and Developmental Biology, Monash University, Clayton, VIC 3800 Australia; 2 Murdoch Childrens Research Institute, Royal Children’s Hospital, Parkville, VIC 3052 Australia; 3 Environmental Futures Research Institute, Griffith University, Nathan, QLD 4111 Australia

**Keywords:** Emu embryo, Limb patterning, Heterochronic, Sonic Hedgehog, Limb bud, Limb development

## Abstract

**Background:**

The forelimb of the flightless emu is a vestigial structure, with greatly reduced wing elements and digit loss. To explore the molecular and cellular mechanisms associated with the evolution of vestigial wings and loss of flight in the emu, key limb patterning genes were examined in developing embryos.

**Methods:**

Limb development was compared in emu versus chicken embryos. Immunostaining for cell proliferation markers was used to analyze growth of the emu forelimb and hindlimb buds. Expression patterns of limb patterning genes were studied, using whole-mount in situ hybridization (for mRNA localization) and RNA-seq (for mRNA expression levels).

**Results:**

The forelimb of the emu embryo showed heterochronic development compared to that in the chicken, with the forelimb bud being retarded in its development. Early outgrowth of the emu forelimb bud is characterized by a lower level of cell proliferation compared the hindlimb bud, as assessed by PH3 immunostaining. In contrast, there were no obvious differences in apoptosis in forelimb versus hindlimb buds (cleaved caspase 3 staining). Most key patterning genes were expressed in emu forelimb buds similarly to that observed in the chicken, but with smaller expression domains. However, expression of *Sonic Hedgehog* (*Shh*) mRNA, which is central to anterior–posterior axis development, was delayed in the emu forelimb bud relative to other patterning genes. Regulators of Shh expression, *Gli3* and *HoxD13*, also showed altered expression levels in the emu forelimb bud.

**Conclusions:**

These data reveal heterochronic but otherwise normal expression of most patterning genes in the emu vestigial forelimb. Delayed *Shh* expression may be related to the small and vestigial structure of the emu forelimb bud. However, the genetic mechanism driving retarded emu wing development is likely to rest within the forelimb field of the lateral plate mesoderm, predating the expression of patterning genes.

**Electronic supplementary material:**

The online version of this article (doi:10.1186/s13227-016-0063-5) contains supplementary material, which is available to authorized users.

## Background

A major question in the field of developmental biology is the relative contribution of changes in gene regulation versus changes in gene structure in generating morphological diversity. The current view of evolutionary developmental biology proposes that diversity is driven by changes in the expression patterns of a common set of deeply conserved genes (the genetic “toolkit”) due to sequence divergence of *cis*-regulatory regions [1–3]. The vertebrate pentadactyl limb is an excellent model system in which to explore this question [4]. The forelimb in particular shows remarkable diversity among vertebrates. Within the avian lineage, the most striking morphological variation of the forelimb is associated with the loss of flight among ratites. Modern ratites (emus, ostriches, rheas, cassowary and kiwi) have lost the ability to fly and have structurally altered or vestigial wing elements [5]. Ratites were once considered a monophyletic group, distributed across the world through vicariance following the breakup of Gondwanaland. However, recent molecular analyses show that extant ratites are polyphyletic, most having evolved the loss of flight independently through dispersal followed by convergence [6–8]. This is consistent with the fact that the forelimb structures of living ratites vary significantly. The rhea and ostrich have well-developed forelimbs (wings) but with reduced distal elements compared to carinate (flying) birds, while in the kiwi, cassowary and emu, all elements are reduced in size and only a single digit is present [9]. In the extinct ratites, the elephant bird and the moa, forelimb structures were different again, with a major reduction in limb skeletal elements in the elephant bird, and a complete absence of wings in moa. Phylogenetic studies, together with the comparative anatomy, suggest that at least three different genetic mechanisms may mediate forelimb development in the different ratite groups. Understanding these mechanisms will shed light on questions of evolutionary developmental biology.

Changes in the pattern of gene expression during embryogenesis must underlie the divergent morphology of ratite wings. However, the molecular basis of vestigial forelimb development among these birds is unknown. Limb morphogenesis has been extensively studied in the chicken embryo, which has a typical avian forelimb structure, comprising well-developed skeletal elements and three digits. A complex interacting network of gene expression results in patterning the chicken forelimb bud in three axes: anterior–posterior, dorsal–ventral and proximal–distal [10–12]. Each of these has a key signaling center that directs differentiation along its axis and integrates genetic information from the other axes. Growth and patterning along the proximal–distal (P–D) axis is largely driven by fibroblast growth factors derived from the apical ectodermal ridge (AER), an epithelial thickening of the limb bud [13–16]. Dorsal–ventral polarity involves antagonism between dorsally secreted WNT growth factors and ventrally expressed *engrailed*-*1* gene. The most finely studied axis is the anterior–posterior (A–P) axis, which is controlled by secretion of the morphogen, *Sonic Hedgehog* (*Shh*). *Shh* is a major player in limb bud growth and development, in both chicken and mammals [17]. It is produced in the posterior region of both fore- and hindlimb buds, demarcating the so-called zone of polarizing activity (ZPA). In a classical morphogen gradient, Shh binds to its receptor, Patched-1 (Ptc1), and regulates expression and proteolytic cleavage of the Gli transcription factors. These factors pattern the anterior–posterior axis of the limb bud, responsible for the number and identity of digits, together with skeletal patterning in the zeugopod (mid region). Shh also maintains expression of *fibroblast growth factor*-*8* (*Fgf8*) in the AER and establishes an auto-feedback loop between the AER and ZPA, coordinating patterning of the A–P and P–D axes [18, 19]. Targeted deletion of *Shh* in the mouse embryo results in truncated limb buds at the zeugopod–stylopod boundary, and a single distal digit [20]. The same phenotype is seen in mouse mutants lacking the deeply conserved long-range *cis*-enhancer of *Shh*, the *ZRS* (ZPA regulatory sequence) [21]. In the chicken, mis-expression of *Shh* in the limb bud induces digit anomalies consistent with its role as an anterior–posterior organizer [22]. Genes 5′ in the *HOXD* cluster also play a role in anterior–posterior patterning of the limb bud (reviewed in [23]).

To shed light on the molecular basis of vestigial forelimb development in the emu, key patterning genes were examined during development. The emu forelimb shows heterochronic development relative to that in chicken, as the developing forelimb bud is small in size and fails to grow into a typical avian wing. Although it does exhibit mesenchymal condensations demarcating the three avian digits, only a single digit (III) is present at hatching and in adults [9]. We found that emu forelimb buds do not proliferate to the extent of hindlimb buds during early bud outgrowth. However, patterning genes were expressed in emu embryonic forelimb buds with profiles comparable to that in chicken, but in a smaller domain. The notable exception was *Shh*. Expression of *Shh* was delayed in emu forelimb buds compared to other patterning genes at the same developmental stage and compared to stage-matched chicken embryos. Meanwhile, the negative regulator of *Shh*, *Gli3*, was up-regulated and the positive regulator, *HoxD13*, was down-regulated, in emu forelimb buds. This suggests that the molecular changes associated with vestigial wing development in emu could involve altered regulation of *Shh* signaling. These data show that most key patterning genes are still expressed in the rudimentary emu forelimb, although Shh appears delayed, representing a heterochronic shift in expression. Changes to the expression pattern of *Shh* have been identified in other vertebrates with divergent limb structure [24], indicating that this gene might be particularly amenable to evolutionary plasticity and that it may act as an important mediator of limb diversity.

## Methods

### Emu embryos

One hundred fertile eggs of the emu (*Dromaius novaehollandiae*) were obtained from a commercial breeder located in rural Victoria, Australia, during the 2013 and 2014 breeding seasons. Eggs were obtained under a Victorian Department of Sustainability and Environment permit number 10005896. Fertile chicken eggs were obtained from Research Poultry Farm, Victoria. All embryos were used under Murdoch Childrens Research Institute Animal Ethics number A694. Eggs were incubated at 37.8 °C and at 40% humidity. Emu embryos were staged according to the morphological criteria of Hamburger and Hamilton [25], originally described for chicken and recently validated for emu embryos [26]. Nagai et al. [26] noted that, proportional to its total incubation time, an emu embryo takes approximately 2–3 times longer to reach an equivalent chicken stage. As emu forelimb growth is retarded, embryos were staged using morphological development of the hindlimb and head/facial characters. Embryos were harvested over developmental stages 18–33 (days 7–25), with stages 19–20 marking the onset of emu forelimb bud outgrowth.

### Cell proliferation and apoptosis

To determine whether reduced development of the emu forelimb bud involved altered cell proliferation of apoptosis, limb buds were immunostained for expression of phospho histone 3 (PH3), a proliferation marker, and cleaved caspase-3 (CC3), an apoptosis marker. At least three embryos from three stages were examined, during early (stages 18, 21) and later (stage 23) bud outgrowth. Forelimb and hindlimb buds were compared. Whole embryos or limb buds were fixed briefly in 4% paraformaldehyde, cryoprotected in sucrose, embedded in OCT, cut and subjected to indirect immunofluorescence as described previously [27].

Limbs were counterstained for DAPI, to denote all cells, and PAX7, which marks immigrating muscle cell precursors from the dermomyotome. Cells were counted from several randomly chosen 10-um frozen sections of limb buds, using ImageJ (Fiji). Cells were counted on random sections across several slides that covered the entire limb buds. This was done for at least three different individuals for each of the stages analyzed. Only cells in the limb bud were counted, where fields were drawn around the limb buds, using ImageJ (Additional file 1: Figure S1). The percentage of positive cells as a proportion of all DAPI+ cells was calculated and graphed. Unpaired *t* tests were used to assess statistical significance, using GraphPad.

### Whole-mount in situ hybridization

Whole-mount in situ hybridization using DIG-labeled RNA probes was carried out as described previously [28]. Briefly, chicken or emu embryos were dissected at stages 18, 19, 20, 21 or 23 and fixed overnight in 4% paraformaldehyde in PBTX. Following dehydration in a series of graded methanols, whole embryos were stored at −20 °C prior to rehydration, digestion in proteinase K (10 µg/mL, for 1 h at room temperature), brief re-fixation and then prehybridization overnight at 65 °C. RNA probes were synthesized from chicken cDNA clones, as emu sequence was not initially available. Subsequent RNA-seq showed very high homology between chicken and emu sequences for most genes analyzed. Probes were labeled with digoxigenin-UTP (Roche, Australia) and added to embryos in prehybridization solution overnight at 65 °C with rocking. Following stringency washes in 2 x SSC and 0.2 x SSC, embryos were incubated for 2-3 h in Antibody Blocking Solution (ABS: 1x TBTX containing 2% BSA + 10% sheep serum). Embryos were then incubated overnight at 4 °C in ABS containing alkaline phosphatase-conjugated anti-DIG antibody (Roche, Australia). Embryos were then extensively washed in TBTX + 0.1% BSA, prior to incubation in chromogen (NBT/BCIP in NTMT buffer, pH 9.5, containing 50 mM MgCl_2_). All color reactions were stopped after 2.5 h, and embryos were washed in PBTX and imaged.

### RNA-seq

Forelimbs and hindlimbs were dissected from emu embryos at stages 20–21 (early bud outgrowth). Duplicate samples were taken, with each replicate comprising six limb bud pairs. Staged-matched chicken embryonic forelimbs and hindlimbs were also harvested. A total of eight samples were generated (2× emu forelimb, 2× emu hindlimb, 2× chicken forelimb and 2× chicken hindlimb). Total RNA was extracted using the QIAGEN RNeasy kit with on-column DNase treatment. RNA was poly-A selected and subjected to deep sequencing on a Hi-Seq 2000 machine (AGFR in Melbourne, Australia). Approximately 45 million, one hundred base pair, paired end reads were obtained per sample. The RNA-seq reads were cleaned using Trimmomatic [29]. Leading and trailing bases with a Phred score below 20 were trimmed. Resulting reads shorter than 50 bp were removed. Cleaned chicken reads were then mapped to the chicken genome (galGal4) using Tophat2 [30], and the number of reads overlapping Ensembl genes was counted using featureCounts [31]. The emu genome is not currently publically available, necessitating de novo reconstruction of the limb transcriptome from the RNA-seq data. Emu reads were pooled and assembled using Trinity with default settings [32]. Reads were mapped back to assembled transcripts using Bowtie with parameters ‘- all-x1000’ [33]. Corset was used to cluster transcripts into gene groups and count mapped reads at the gene level [34]. Assembled emu transcripts were annotated using the highest scoring BLAST alignment to chicken genes [35]. Any transcript with an *E* value below 10^−5^ was considered unknown. The gene-level annotation was then determined as the most frequent from among the gene’s transcripts. When multiple emu genes were found to match a single chicken gene, their corresponding counts were aggregated. This arose due to gaps in the assembled sequences. Statistical testing for differential expression was carried out using voom and Limma [36]. Counts from chicken and emu were analyzed together using an experimental design that accounted for both species and limb type. Genes with fewer than 10 counts in 6 or more samples were removed from the analysis. P-values were adjusted for multiple testing using the Benjamini–Hochberg procedure [37].

## Results

### Development of emu forelimb and hindlimb buds

**Fig. 1:**
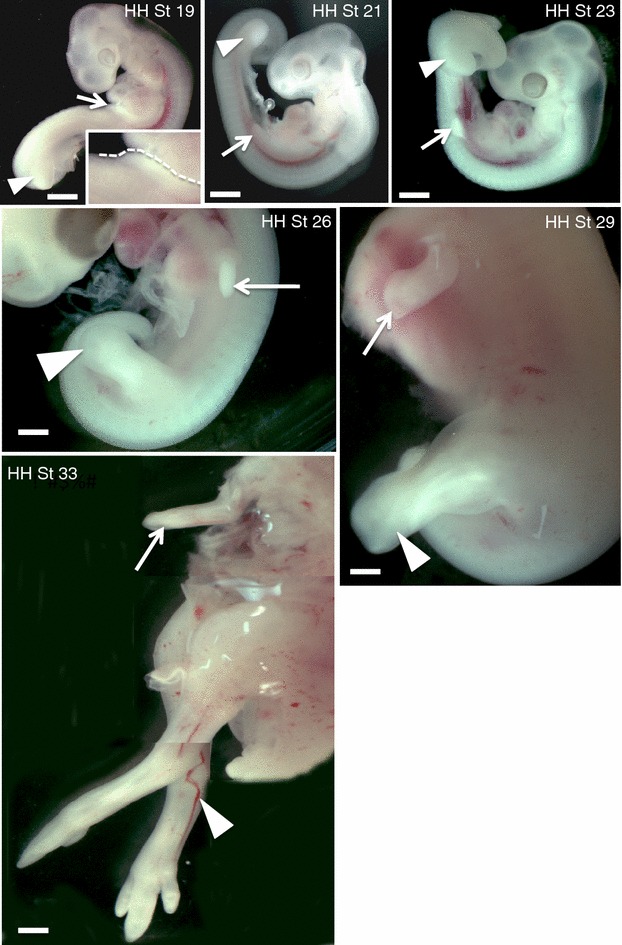
Development of the forelimb and hindlimb buds in emu embryos (staged by hindlimb and head characters). The hindlimb bud appears prior to the forelimb bud. Outgrowth of the forelimb bud is both delayed and retarded. The forelimb bud appears at HH stage 19 (see *insert*), when outgrowth of the hindlimb bud is already advanced. By stage 26, the hindlimb bud is significantly larger than the forelimb bud. By stage 33, the forelimb bud appears thin and elongated, with a single developing digit. The hindlimb buds are greatly elongated as they differentiate into legs and feet. Forelimb bud denoted by *arrows*. Hindlimb bud denoted by *arrowheads*. *Bar* 1 mm

In the chicken embryo, both fore- and hindlimb limb buds first become demarcated from the body wall as small mesodermal bulges at stages 17–18 (Hamburger and Hamilton [25]). In contrast, the forelimb and hindlimb buds of emu embryos show heterochronic growth. Nagai et al. [26] reported that the hindlimb buds appear in the emu embryo at HH stages 17–18, as in chicken. However, forelimb bud development is delayed, first appearing at 20 [26]. In the study described here, the forelimb bud first became apparent at stage 19, when the hindlimb bud was already more advanced (Fig. 1). Outgrowth of the hindlimb bud continued during stages 20–26, while growth of the forelimb was significantly slower (Fig. 1). By stage 29, the hindlimb buds were differentiating into distinct stylopod, zeugopod and autopod elements, while the forelimb buds were club-like. By stage 33, the emu forelimbs became thin and elongated, with a single digit developing (Fig. 1). These observations suggest that the molecular signals involved in forelimb bud outgrowth in emu differ from those in the hindlimb bud.

### Reduced cell proliferation in emu forelimb versus hindlimb buds

**Fig. 2:**
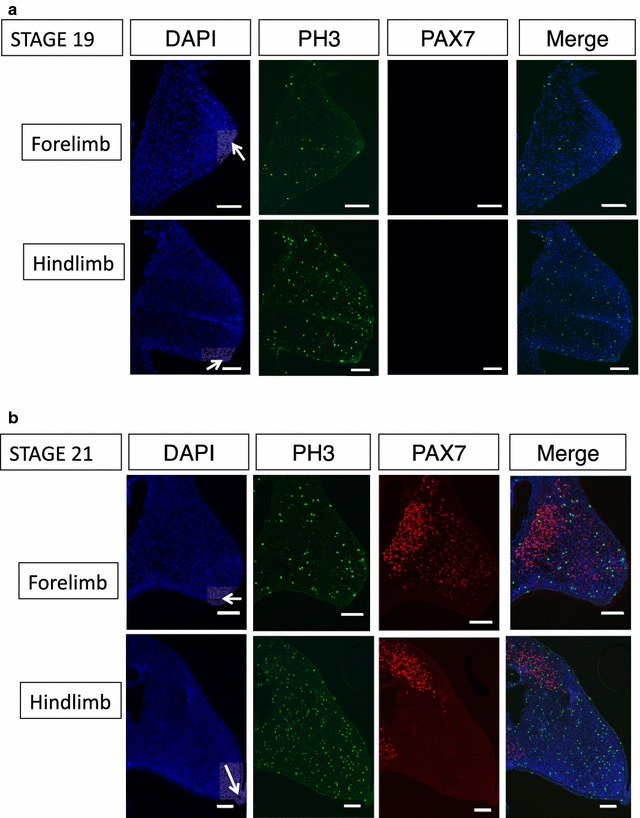
Growth of emu limb buds and cell proliferation at HH stage 19 (**a**) and stage 21 (**b**). Transverse sections. The apical ectodermal ridge (AER) is indicated by a *white arrow* in DAPI-stained sections. *Green* PH3 expression (a marker of cell proliferation); *red* PAX7 expression. There are fewer proliferating cells in the forelimb bud, and those that are proliferating are not immigrating PAX7+ cells. *Bar* 100 µm

**Fig. 3:**
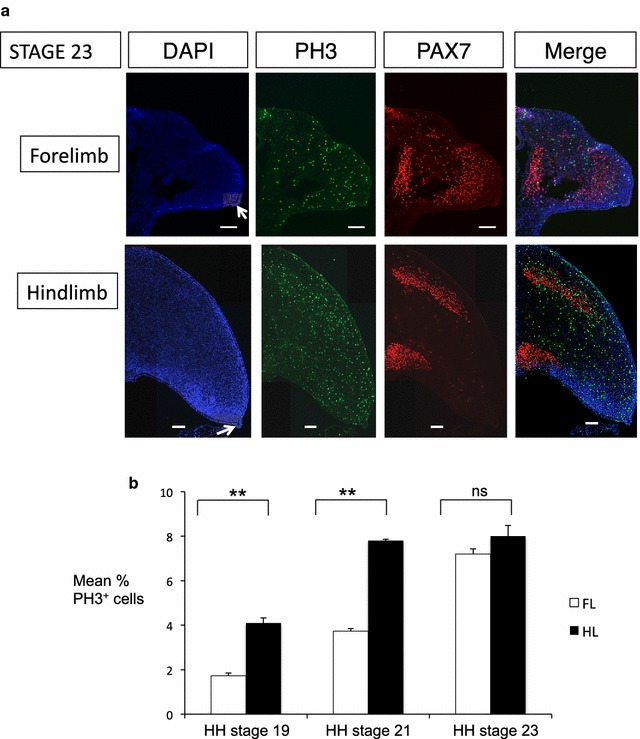
**a** Cell proliferation in emu limb buds at HH stage 23. Transverse sections. The apical ectodermal ridge (AER) is indicated by a *white arrow* in DAPI-stained sections. *Green* PH3 expression (a marker of cell proliferation); *red* PAX7 expression. In the emu forelimb bud, PAX7+ cells migrate as a cloud, while those in the hindlimb bud migrate into distinct domains. *Bar* 100 µm. **b** Quantification if cell proliferation. Mean number of PH3^+^ cells as a percentage of total DAPI^+^ cells in forelimb versus hindlimb buds over stages 19, 21 and 23. Cells were counted using Fiji software over several randomly sampled transverse sections. Data show mean ± SEM, *n* = seven (**p < 0.01; *ns* not significant)

To examine proliferation, limb buds were examined at stages 19, 21 and 23. At stage 19, the limb forelimb bud was only just visible macroscopically, but the hindlimb bud was more advanced. In early-stage emu embryos, when the forelimb bud was just emerging (HH 19 and 21), there were fewer proliferating cells in the forelimb bud compared to the hindlimb bud, as assessed by PH3 immunofluorescence. At HH stage 19, there was an average of 2% proliferating cells in the emu forelimb bud versus 4% in the hindlimb bud (*p* = 0.009; unpaired *t* test). Similarly, at HH stage 21, there were significantly fewer proliferating cells in the forelimb bud versus the hindlimb bud (4 and 7%, respectively, *p* = 0.004; unpaired *t* test) (Figs. 2, 3). By stage 23, proliferation rates were comparable between the two buds (approximately 8%) and not significantly different (*p* = 0.08) (Fig. 3). Sections of limb buds were also assayed for apoptosis, using cleaved caspase-3 as the marker. There were very few apoptotic cells (less than 1% in both fore and hindlimb buds) and no significant differences between the two limb buds (*p* > 0.08) (Additional file 2).

PAX7 was used as a marker to identify muscle cell precursors migrating into the limb buds which can be detected from at least as early as stage 21. These immigrating cells were almost all PH3 negative, indicting that the difference in cell proliferation between the fore- and hindlimb buds likely derives from resident mesenchymal cells (presumptive chondrocytes and connective tissue). Small numbers of proliferative cells were present in the AER of both fore- and hindlimb buds at each stage examined, with no differences between buds (Figs. 2, 3). Interestingly, the immigrating PAX7+ cells showed some differences in their distribution between the forelimb and hindlimb buds. The cells migrated into the forelimb bud as a “cloud” but resolved into discrete domains in the hindlimb bud, corresponding to the position of the major tricep and bicep muscle masses (Figs. 2, 3).

### Expression of key limb patterning genes in emu embryos

**Fig. 4:**
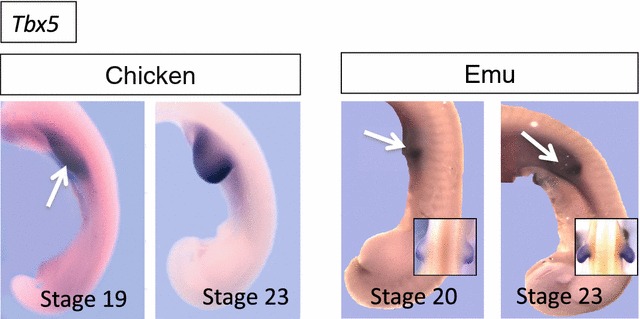
Expression of the *Tbx5* gene in emu versus chicken limb buds (HH stages 18–22). *Arrows* shows forelimb. For emu *Tbx5*, *insets* show higher magnification dorsal views

**Fig. 5:**
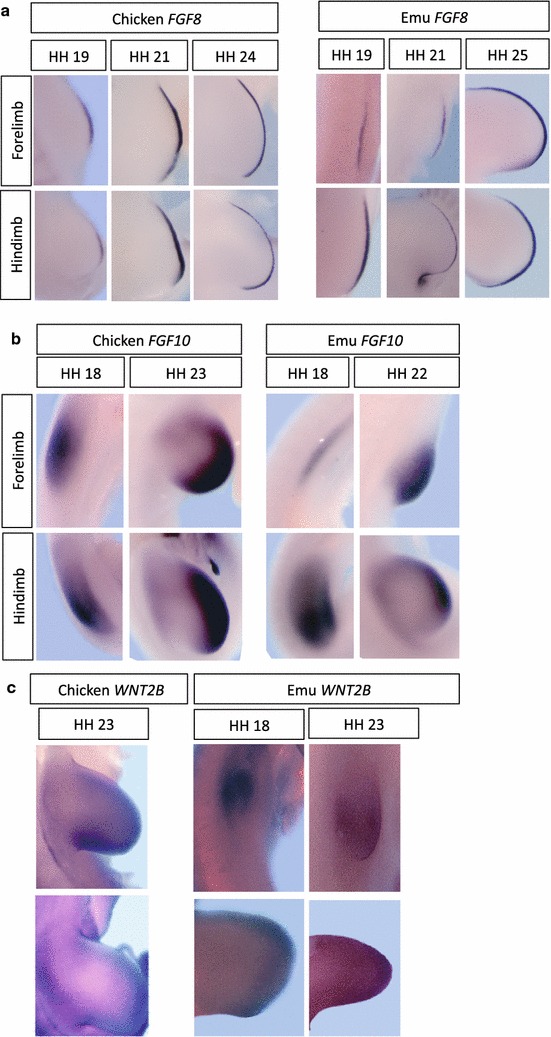
Expression of genes associated with limb bud outgrowth (*Fgf10*, *Fgf8*, *Wnt2b*) in emu versus chicken embryos (stages 18, 19, 22 and 23). **a** Fgf8 is expressed in the AER of both species, but in a smaller domain in emu forelimb bud, due to the delayed growth and smaller size of the limb bud. **b**, **c** Similarly, Fgf10 and Wnt2 show reduced domains in the emu forelimb bud

To investigate the molecular mechanism of vestigial forelimb development in emu embryos, key limb patterning genes were studied at the time of bud initiation and outgrowth (stages 18–23). In chicken and mouse embryos, the fore- and hindlimb buds derive from lateral plate mesoderm (LPM) in response to the combinatorial *Hox* code along the anterior–posterior axis of the embryo. Initiation of the forelimb bud is regulated by the critical T-box transcription factor, *Tbx5* [38, 39]. Tbx5, in turn, regulates expression of *Fibroblast growth factor 10* (*Fgf10*) in the LPM. Fgf10 then initiates cell proliferation in the LPM and induces expression of *Fgf8* in the overlying ectoderm, giving rise to the critical apical ectodermal ridge (AER). In the chicken embryo, the signaling molecule, Wnt2b, has been shown to act upstream of *Tbx5*, before the establishment of positive feedback loops between *Tbx5*, *Fgf10* and *Wnt2b* to coordinate forelimb bud outgrowth [40]. These genes are highly conserved and are essential for the emergence of forelimb buds in both chicken and mouse embryos (reviewed in [41]). In the study reported here, expression of these genes in emu embryos was compared to that in the chicken. In emu embryo, *Tbx5* was expressed in the forelimb field, as in chicken, albeit with a smaller expression domain, as assessed by whole-mount in situ hybridization (Fig. 4). Emu *Wnt2b*, *Fgf10* and *Fgf8* were all expressed in a similar pattern to that observed in stage 19–20 chicken embryos, but again with smaller forelimb domains compared to the hindlimb (Fig. 5). The smaller domain of expression reflected the diminutive size of the emu forelimb bud relative to that in chicken. *Fgf10* and *Wnt2b* mRNAs were both robustly expressed in the mesoderm of the emu forelimb bud, despite its smaller size. In both chicken and emu, *Fgf10* was strongly expressed in the LPM and nascent limb bud in the forelimb field at stage 18 (Fig. 5). As in chicken embryos, *Fgf8* expression was limited to the AER in both fore- and hindlimb buds of emu. These expression patterns show that the emu forelimb bud shares the same key signaling factors established for chicken and mouse, but in a smaller field, reflecting the smaller size of the forelimb bud.

**Fig. 6:**
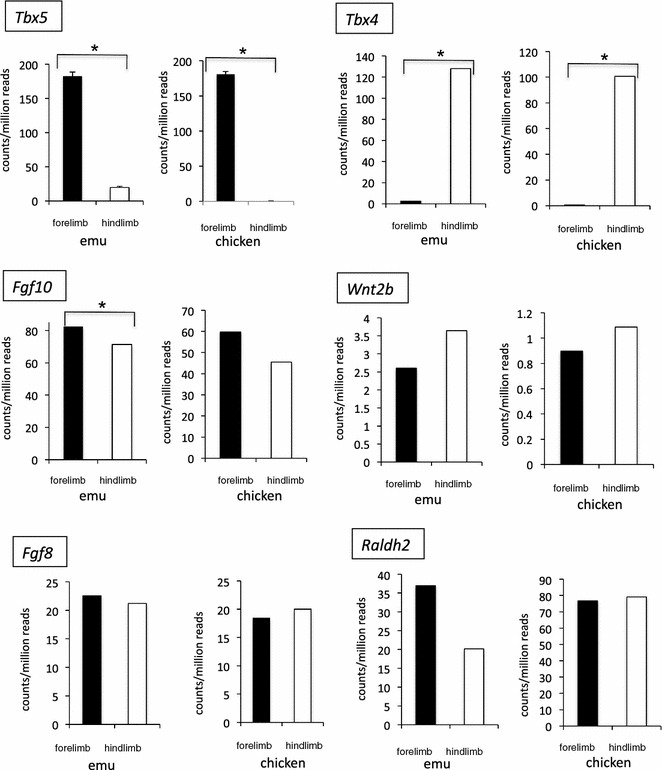
RNA-seq analysis of genes associated with limb bud outgrowth in emu versus chicken embryos (HH stages 20–21). Statistically significant differential expression (adjusted *p* < 0.05) is shown by *asterisks*. *Black* forelimb; *white* hindlimb. *Fgf10* expression was higher in the emu forelimb bud than in the hindlimb bud at the time of outgrowth (stages 20–21)

The results above were confirmed by RNA-seq, which identified all of the mRNAs expressed in emu and chicken limb buds at stages 20–21 (the total RNA-seq data are being made publically available through a second paper on emu forelimb development; Farlie et al., under review). At stage 20/21, chicken forelimb and hindlimb buds are of similar size and shape [25]. At stages 20–21, the emu hindlimb is similar to that in chicken, while the forelimb bud is just initiating outgrowth. Validity of the RNA-seq data was first confirmed by examining the expression of *Tbx5* and *Tbx4*, known forelimb- and hindlimb-specific mRNAs in chicken and mouse. As expected, these transcripts showed strong forelimb- and hindlimb-restricted expression in both species (Fig. 6). RNA-seq confirmed expression of other genes associated with Tbx function. *Fgf10* expression was significantly higher in emu forelimb compared to hindlimb at stage 20/21 (*p* = 0.007), but not significantly higher in chicken (*p* = 0.32), while *Wnt2b* was not differentially expressed between forelimb and hindlimb of either species (*p* = 0.39 is emu and 0.99 in chicken) (Fig. 6). Similarly, *Fgf8* was not differentially expressed between fore- and hindlimb buds in either species (*p* = 0.30 is emu and 0.90 in chicken) (Fig. 6). *Raldh2*, responsible for retinoic acid synthesis during limb bud initiation, was not differentially expressed in either species (Fig. 6). Overall, these data indicate that many key genes associated with early limb bud formation are normally expressed in emu forelimbs buds.

### Delayed Sonic hedgehog expression in emu forelimb buds

**Fig. 7:**
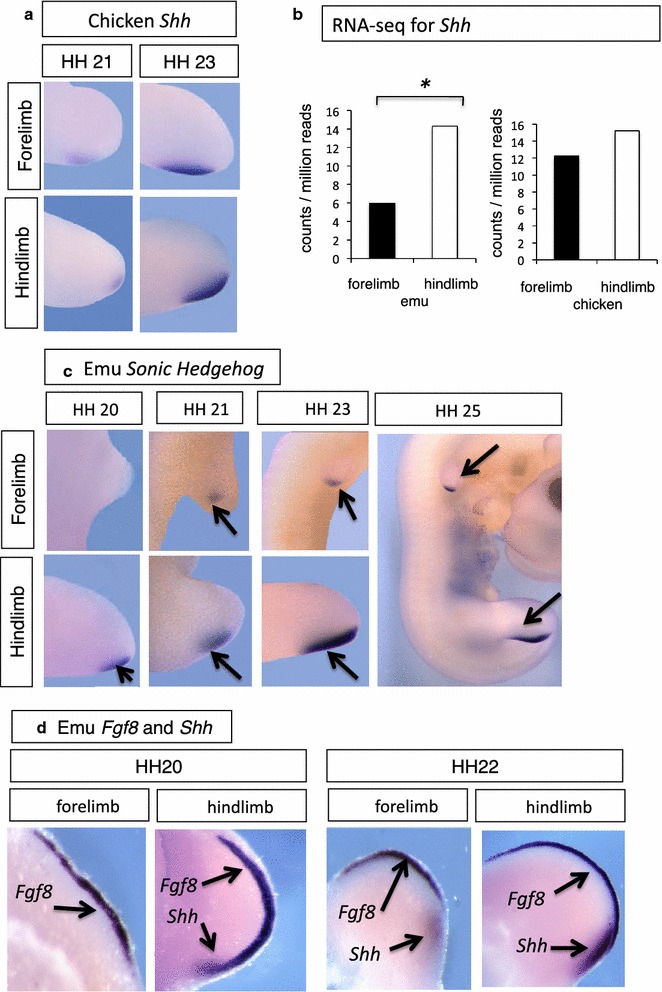
Delayed expression of Sonic Hedgehog (*Shh*) in emu forelimb buds. Whole-mount in situ hybridization and RNA-seq. **a** Equivalent expression of Shh in both forelimb and hindlimb buds of chicken embryo (stages 21 and 23). **b** RNA-seq analysis at stage 20, showing lower level of *Shh* expression emu forelimb bud (stages 20–21) compared to hindlimb buds, but not in the chicken. **c** Delayed *Shh* expression in emu embryo forelimb buds relative to hindlimb buds (HH stages 20–25). *Arrows* show forelimb and hindlimb buds (outlined). **d** Co-localization of *Fgf8* and *Shh* mRNA in stages 20 and 22 emu limb buds, showing robust *Fgf8* expression but lower and delayed expression of *Shh* in forelimb versus hindlimb buds

We next examined expression of *Sonic Hedgehog* (*Shh*), a key organizer of the developing limb (Fig. 7). *Shh* expression at the posterior margin of developing forelimbs and hindlimbs defines the zone of polarizing activity (ZPA), an organizer that directs anterior–posterior patterning and also contributes to maintenance of the AER. In the chicken, *Shh* was expressed as expected at the posterior region of both fore and hindlimb buds at stages 21 and 23 (Fig. 7b). In contrast, in emu embryos, there was a temporal difference in *Shh* expression between forelimb and hindlimb buds (Fig. 5c). At stage 20, emu *Shh* was expressed in the hindlimb but not in the forelimb bud, as assessed by in situ hybridization (Fig. 7c). By stage 21, a small domain of *Shh* expression was detectable in the emu forelimb bud, compared to robust expression in the hindlimb bud. Expression increased in the emu forelimb bud as development proceeded, but was never as strong or extensive as that in the hindlimb bud, which was similar to that in chicken limb buds (Fig. 7c). In the chicken embryo, *Shh* is first detectable in both fore- and hindlimb at the time of bud outgrowth (from stage 17) [42]. However, even accounting for the delayed development of the emu forelimb bud relative to the hindlimb, *Shh* expression was not initially detectable until after the initiation of forelimb bud outgrowth (stage 21; Fig. 7c). This delay in expression was also detected by RNA-seq, which showed a lower level of Shh expression in the forelimb bud versus the hindlimb bud at stage 21 (*p* = 0.001) (Fig. 7b). Thereafter, *Shh* expression was low but detectable in the emu forelimb bud. Other key patterning genes, such as *Tbx5*, *Fgf10*, *Fgf8* and *Wnt2b*, were all robustly expressed in the emu forelimb bud over this period, despite its smaller size (Fig. 6), and hence the delayed expression of emu *Shh* in the forelimb, relative to the other genes, was unusual.

Double in situ staining was carried out for *Shh* and *Fgf8* expression in emu embryos. These two genes show mutual positive regulation in chicken and mouse. At the time of forelimb bud initiation (stage 20), emu *Fgf8* was expressed in the absence of detectable *Shh* expression, while both genes were expressed in the hindlimb bud (Fig. 7d). At stage 22, low *Shh* expression could be detected in the growing forelimb bud, while strong expression of both *Shh* and *Fgf8* was detected in the emu hindlimb bud (Fig. 7d). Hence, at stages 20–21, when *Shh* regulators such as *Tbx5* and *Fgf8* are robustly expressed, *Shh* itself is more lowly expressed in the emu forelimb bud.

**Fig. 8:**
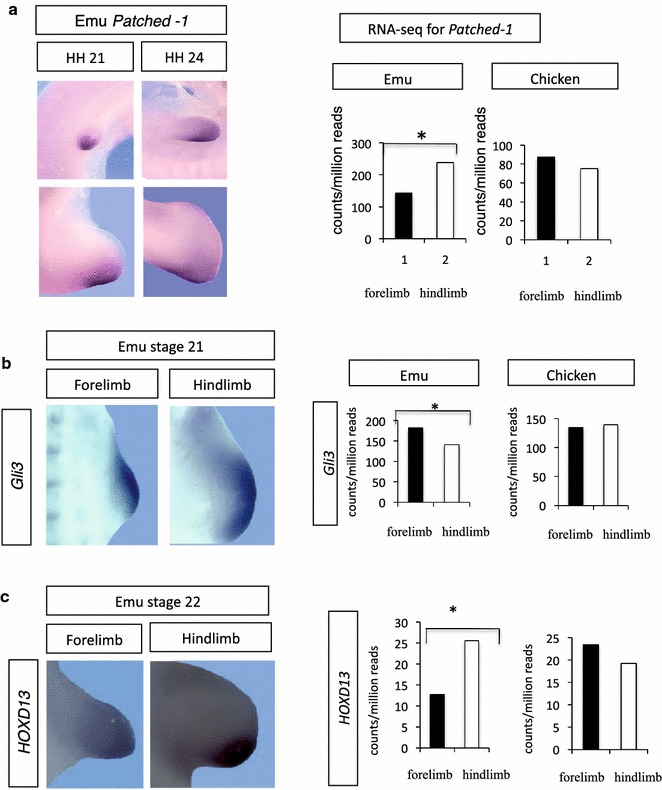
Gene expression in emu versus chicken limb buds; *Patched-1*, *Gli3 and HoxD13*. **a** Expression of *Patched*-*1* mRNA in emu limb buds, at stages 21 and 24. Localization is shown by in situ hybridization; levels are shown by RNA-seq (with chicken as control). A lower level of Patched-1 expression was observed in the emu forelimb bud. **b** Expression of *Gli3* mRNA expression in emu forelimb buds (stage 21). Localization is shown by in situ hybridization; levels are shown by RNA-seq (with chicken as control). *Gli3* expression was elevated in emu forelimb bud versus the hindlimb bud at stages 20–21. **c** Expression of *HoxD13* in expression in emu forelimb buds (stage 21). Localization is shown by in situ hybridization; levels are shown by RNA-seq (with chicken as control). *HoxD13* expression was lower in emu forelimb bud versus the hindlimb bud

Other components of the *Shh* signaling pathway were examined in emu and chicken embryonic limb buds. In situ hybridization showed that the Shh-dependent receptor, *Patched*-*1*, was expressed with a posterior bias in both the forelimb and hindlimb buds of emu embryos at stages 21 and 23 (Fig. 8). However, *Patched*-*1* expression was lower in the emu forelimb bud at stages 20–21, as assessed by RNA-seq (*p* = 0.0001) (Fig. 6). This may reflect the earlier stage of development of the forelimb relative to the hindlimb bud. Gli3 is a *Shh* antagonist and initially restricts *Shh* expression to the ZPA, but subsequently comes under Shh control. *Gli3* mRNA was expressed with an anterior bias in emu fore- and hindlimb buds, while RNA-seq revealed elevated *Gli3* mRNA in the forelimb compared to the hindlimb of emu but not chicken embryos at stage 20/21 (*p* = 0.0001; Fig. 8). The transcriptional activator of *Shh*, *Hand2*, was weakly expressed but at similar levels in both emu and chicken limb buds at the same stage (not shown). *HoxD13*, a posterior regulator, was expressed with posterior bias in emu limb buds, as in other vertebrates (Fig. 8c). However, RNA-seq revealed that *HoxD13* was more weakly expressed in stage 20/21 emu forelimb compared to hindlimb buds (*p* = 0.010) (Fig. 8c). *Hoxd12* was more highly expressed in chicken stage 20/21 forelimb compared to hindlimb bud (*p* = 0.001), but was expressed at similar levels in emu limb buds (not shown). The proposed negative regulators of *Shh*, *Etv4* and *Etv5* were not differentially expressed in emu forelimb versus hindlimb buds, nor were *Wnt7a*, *Lmx1b* and *Smad 1*, *5* and *8* (not shown).

**Fig. 9:**
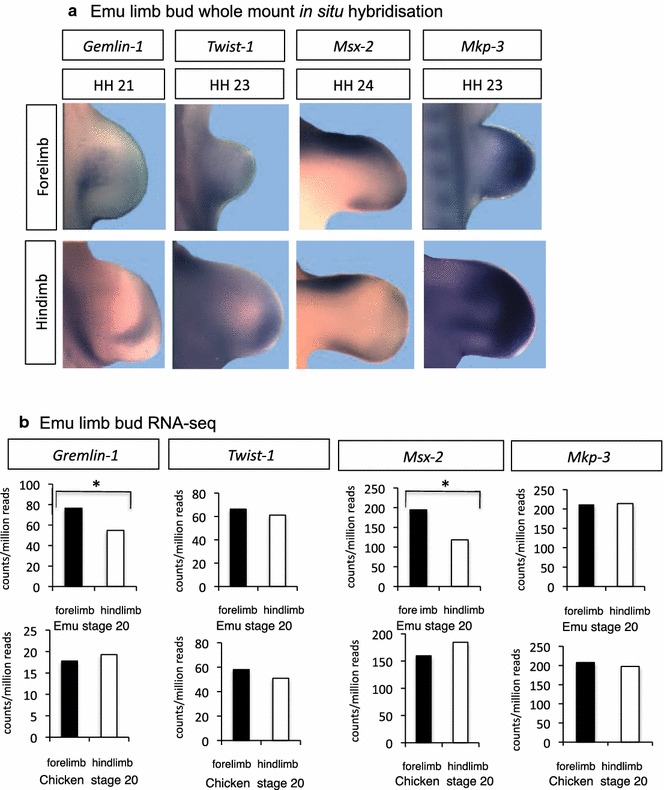
Expression of *Gremlin*, *Twist1*, *Msx*-*2* and *Mkp*-*3* in emu and chicken limb buds. **a** Whole-mount in situ hybridization shows localization in limb buds. **b** RNA-seq shows relative expression levels in emu versus chicken at stages 20–21. Adjusted *p* < 0.05 denoted by *asterisks*). Both Gemlin-1 and Msx-2 showed elevated expression in emu forelimb buds relative to hindlimb buds at stages 20–21

In chicken and mouse, *Shh* up-regulates the BMP antagonist Gremlin, which affects BMP regulation of the AER. *Gremlin* was expressed in the posterior region of both forelimb and hindlimb buds of stage 21 emu embryos, and expression was higher in the forelimb bud (*p* = 0.003) (Fig. 9). The putative negative regulator of Shh, *Twist*-*1*, was expressed at similar levels in both forelimb and hindlimb buds, while the homeobox gene, *Msx*-*2*, showed a distinct spatial expression pattern in the anterior region of both fore- and hindlimb buds (Fig. 9). RNA-seq showed that *Msx*-*2* was more highly expressed in the emu forelimb bud compared to the hindlimb (*p* = 0.0008), in comparison with the comparable expression between fore- and hindlimbs in chicken (*p* = 0.316).

## Discussion

The emu has a remarkably divergent wing structure related to the loss of flight and a terrestrial existence. The bony elements of the emu wing are all greatly reduced relative to body size. The humerus, radius, ulna and metacarpals are all reduced, while the typical avian three-digit arrangement is replaced by a single claw-bearing digit (corresponding to embryonic digit III) [5]. However, during emu embryonic development, mesenchymal condensations corresponding to digits II, III, IV and V develop, transiently expressing the chondrogenesis marker, *Sox9* [9]. However, only emu forelimb digit III persists and undergoes ossification and develops (whereas digits II, III and IV ossify in chicken). The stunted forelimb of the emu is patterned very early in development and is marked by heterochronic forelimb bud outgrowth. The forelimb bud is delayed relative to the hindlimb bud, and it grows at a slower rate and lacks typical avian three-digit differentiation (Figs. 1, 2, 3). Furthermore, the migration of PAX7+ cells into the emu forelimb bud is atypical for avians (Figs. 2, 3). The expression of limb bud outgrowth genes, *Tbx5*, *Fgf10*, *Wnt2b* and *Fgf8*, is similar between chicken and emu at stages 18–20 and 23 (Figs. 4, 5). These observations indicate that the vestigial development of the emu forelimb is unlikely due to delayed or altered expression of these key genes.

Heterochronic growth of the emu forelimb bud appears to be initiated early in development, in the lateral plate mesoderm forelimb field, since the forelimb bud outgrowth lagged behind that of the hindlimb bud from the earliest stages of development (Figs. 1, 2). Consistent with this observation, we noted a marked reduction in proliferation at the time of forelimb bud initiation (relative to hindlimb), while this difference was not evident at later stages. The driver of this reduced proliferation in the emu is unclear, since Fgf10, which is a key regulator of proliferation at the initiation stage, was robustly expressed in emu forelimb buds. Previous studies in amphibians have shown that experimental reduction in cell proliferation in the limb bud can result in small limbs that lose skeletal elements [43]. In the emu embryo, vestigial wing development appears to depend upon reduced numbers of mesenchymal cells in the limb field. Reduced wing bud proliferation may involve changes to the expression patterns of genes regulating the initiation of limb bud outgrowth, such as retinoic acid synthesis and action and *Hox* gene expression in the flank, and is a potential avenue for future research. Altered proliferation in the forelimb bud appears to be a key driver of differential outgrowth since we did not observe any differences in cell death between fore- and hindlimb buds (Additional file 2).

Among the major players in limb patterning, *Shh* showed delayed expression in the emu forelimb bud, relative to other key genes, such as *Fgf8*. *Shh* plays two major roles in patterning the limb bud. Operating within the ZPA signaling center, *Shh* coordinates the genetic network required for proper limb morphogenesis (maintaining *Fgf8* in the AER, for example). It also establishes the crucial anterior–posterior axis of the developing limb, by antagonizing the Gli3-truncated transcriptional repressor (Gli3R). Targeted deletions in mouse show that *Gli3* restricts, while *Shh* expands, digit number [44]. Overall limb morphogenesis and anterior–poster patterning in particular are strongly influenced by *Shh*, and both of these processes are altered in the emu forelimb, which is small and thin and has only a single digit. A delay in *Shh* expression could be expected to impact patterning of the zeugopod and autopod, resulting in reduced digit number. Alternatively, Shh has also been shown to impact digit number through alteration to proliferation, independently of its overt patterning influence [45]. In the embryos of odd and even toed mammals, digit loss can be due to genetic mechanisms operating during limb bud patterning (Shh mediated reduction in *Patched* expression without cell death in the cow and pig) or during the post-patterning phase (expanded domains of apoptosis in the horse and jerboa). Hence, different mechanisms can operate to regulate digit loss [46, 47]. Emu embryos exhibit mesenchymal condensations typical of avian forelimb buds, but most fail to ossify. In the chicken, the *oligozeugodactyly* mutant (*ozd*) lacks *Shh* function in the limb, resulting in loss of *Patched*-*1* and *Gli1* expression. The forelimb bud exhibits elevated cell death and becomes thin, narrow and sickle-shaped, distal elements are hypoplastic and digits are absent, while the hindlimb develops a single digit [48]. This phenotype derives from a deletion within the *ZRS* regulatory region of chicken *Shh* [49]. Similarly, in the emu embryo, the divergent structure of the forelimb is correlated with altered Shh signaling, although there does not appear to be any elevation in cell death, raising the possibility of a different mechanism for digit loss.

Delayed *Shh* expression in the emu forelimb bud may be due to novel expression patterns of a trans-regulatory factor/s, or changes in *Shh* 5′ regulatory sequences. We examined expression of candidate or known upstream regulatory factors, using in situ hybridization and RNA-seq. In the developing vertebrate limb bud, *Hand2* and 5′ *Hoxd* genes are responsible for transcriptional activation of *Shh* [50–52]. Prior to *Shh* expression, mutual antagonism between the transcription factors, Gli3 and Hand2, along with the *Hox D11*–*13* genes, polarizes the limb bud across the anterior–posterior axis [53]. Gli3 restricts expression of *Hand2* to the posterior limb margin, while *Hand2* restricts expression of *Gli3* to the anterior region. Hand2 activates *Shh*. By restricting *Hand2* expression to the posterior pole, Gli3 indirectly limits *Shh* expression to the ZPA [19]. The mechanism of delayed *Shh* expression in emu forelimb could therefore be related to the elevated *Gli3* detected by RNA-seq (Fig. 8). Indeed, an expanded anterior *Gli3* domain in the embryonic emu forelimb was reported by de Bakker et al. [9] using in situ hybridization. This same study examined other patterning genes, including *Shh*, but did not report a delayed expression of *Shh* in emu limb buds. However, only one stage was reported (stage unclear) [9].

RNA-seq showed that *Hand2* expression was similar in emu forelimb and hindlimb buds at stage 20/21 (not shown). However, *Hand2* mRNA could be spatially restricted in the presence of elevated *Gli3*, hence retarding *Shh* in the forelimb bud. Spatial expression of *Hand2* was not examined here, but is worth further investigation. With respect to *Hox* genes, De Bakker et al. [9] reported that the early posterior expression domains of *HoxD11* and *HoxD12* are conserved in emu forelimbs. Similarly, we detected comparable levels of *HoxD11* and *HoxD12* mRNA in emu fore- and hindlimb buds (not shown). However, we did detect a significantly lower level of *HoxD13* expression in emu forelimb relative to the hindlimb at stages 20–21 (Fig. 8). However, this may reflect the earlier developmental stage of the forelimb relative to the hindlimb bud and requires further analysis. The developmental basis of delayed Shh signaling in the emu forelimb bud might therefore be traced back to a common upstream regulator of *Hoxd13* and *Gli3* expression, such as retinoic acid (RA). However, enzymes associated with RA biosynthesis, such as *Raldh2*, were not differentially expressed in emu limb buds (Fig. 3).

The alternative mechanism of divergent *Shh* expression in emu forelimb buds would involve mutational changes to its regulatory region. The long-range regulator of *Shh* expression in the limb bud is the *ZRS*, a highly conserved 780-bp sequence located within the fourth intron of the widely expressed *Lmbr1* gene [54, 55]. Mutations within the *ZRS* affect the spatial and temporal expression profile of *Shh*. Natural or induced point mutations in *ZRS* are sufficient to induce aberrant *Shh* expression and limb dysmorphologies in mice and humans [56] reviewed in [57]. Most recently, it has been shown that loss or degradation of the ZRS plays a critical role in the evolution of limb loss in snakes [58]. It is known that different *Hox* genes influence *Shh* expression differently in the forelimb versus the hindlimb. For example, the *Hox9* paralogues (*HoxA*–*HoxD9*) regulate *Hand2* expression, and hence *Shh*, while *Hox5* paralogues appear to maintain *Shh* expression at the ZPA, but only in the forelimb bud (reviewed in [59]). Hence, by extension, it is possible that mutations in emu ZRS could affect *Shh* expression in the forelimb and hindlimb bud differently. Recent deletion analysis of the mouse *ZRS* has shown that it comprises two distinct but integrated bipartite domains, which are interpreted differently in the forelimb and hindlimb buds [60]. In the absence of the second domain, there is no *Shh* expression in the forelimb. The dominant preaxial polydactyly locus (*Po*) in the Silkie breed of chickens is attributed to a single nucleotide polymorphism in the *ZRS*, resulting in expanded and prolonged *Shh* expression in developing limb buds and an extra leg digit [61]. Hence, subtle changes in ZRS sequence can drive quite divergent spatiotemporal expression patterns of *Shh* that result in divergent morphologies. An examination of the ZRS among ratites might therefore be of interest.

The Sonic Hedgehog signaling pathway may be particularly amenable to evolutionary change, acting as a major source of developmental plasticity in the limbs of vertebrate embryos [62]. In the developing bat forelimb, a second wave of *Shh* expression occurs in the interdigital region, which is thought to underlie the unique morphogenesis of the forelimb (wing) [24]. In dolphins, which lack patent hindlimbs, the hindlimb bud degenerates during embryogenesis. An AER initially forms in the dolphin hindlimb bud, and Fgf8 expression is initiated, but there is a total loss of *Shh* expression, leading to lack of AER maintenance and limb bud regression [63]. Similarly, temporal shifts in *Shh* expression during limb formation cause changes in digit number in some lizard species [64]. *In* the marsupial, *Monodelphis*, forelimb growth is advanced and corresponds to an early *Shh* expression pattern [65]. These diverse examples all implicate *Shh* signaling as a developmental hub involved in the variation seen in limb morphologies.

In chicken and mouse embryos, *Shh* expression at the anterior region of the limb bud is antagonized by *Msx*-*2* expression, [66]. Msx-2 was up-regulated in the early emu forelimb bud (Fig. 9). Hence, delayed forelimb *Shh* expression observed here correlates with elevated expression of *Msx2*. *Msx*-*2* is a suppressor of morphogenesis in the mesoderm of developing limb buds, and over-expression causes extensive cell death. Interestingly, this results in chicken embryos with long, narrow limbs [67], broadly similar in morphology to that observed in the emu forelimb. This warrants further study.

## Conclusions

This study describes heterochronic development of the forelimb and hindlimb buds of the emu embryo, which has a vestigial wing structure at hatching. The small emu limb bud exhibits reduced cell proliferation among resident mesenchymal cells at early stages. All major limb patterning genes are expressed in the emu forelimb bud, albeit with a reduced expression domain due to the smaller size of the forelimb bud. However, expression of *Shh* is delayed in the emu forelimb bud compared to other key genes. Negative and positive *Shh* regulators, *Gli3* and *HoxD13*, are up- and down-regulated, respectively, at the time of forelimb bud outgrowth (stages 20–21). Changes in the expression patterns of these three genes may play a role in the divergent structure of the emu forelimb bud. However, the exact molecular mechanism driving the formation of the vestigial emu wing is still unclear. Lower rates of cell proliferation early in development point to a mechanism that operates at the time of forelimb field specification in the lateral plate mesoderm, preceding expression of limb patterning genes.

## Additional files

**Additional file 1.** Cell counting using ImageJ (Fiji). Stage 21 emu forelimb bud is shown for example.

**Additional file 2.** Staining for apoptosis in embryonic emu limb buds. Immunofluorescent detection of Cleaved Caspase 3 (CC3). Very few apoptotic cells are detected in forelimb and hindlimb buds. Data for stage 21 is shown. Results were the same for other stages.
