# Supplementary figures and images for: Limb patterning genes and heterochronic development of the emu wing bud
Source: EvoDevo. 2016 Dec 20;7:26. doi: 10.1186/s13227-016-0063-5 (PMC5168868; doi:10.1186/s13227-016-0063-5)

Cell counting using ImageJ (Fiji).  
Stage 21 FL is shown for example.

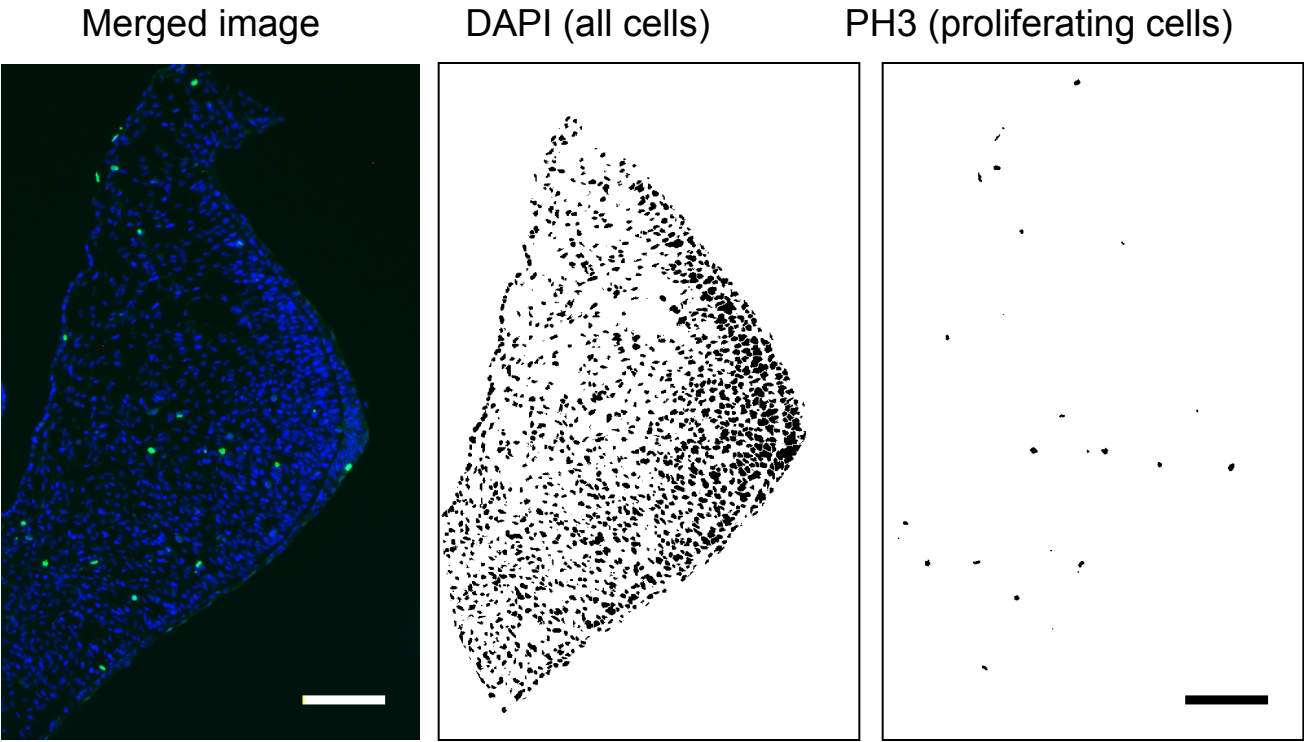

Supplement: Supplementary file 1 — Additional file 1. Cell counting using ImageJ (Fiji). Stage 21 emu forelimb bud is shown for example. [file 13227_2016_63_MOESM1_ESM.pdf]
