# Supplementary material for: Limb patterning genes and heterochronic development of the emu wing bud
Source: EvoDevo. 2016 Dec 20;7:26. doi: 10.1186/s13227-016-0063-5 (PMC5168868; doi:10.1186/s13227-016-0063-5)

Smith et al.

Suppl. Figure X. Staining for apoptosis in embryonic emu limb buds. Immunofluorescent detection of Cleaved Caspase 3 (CC3). Very few apoptotic cells are detected in forelimb and hindlimb buds. Only stage 21 is shown. Data were the same for other stages.

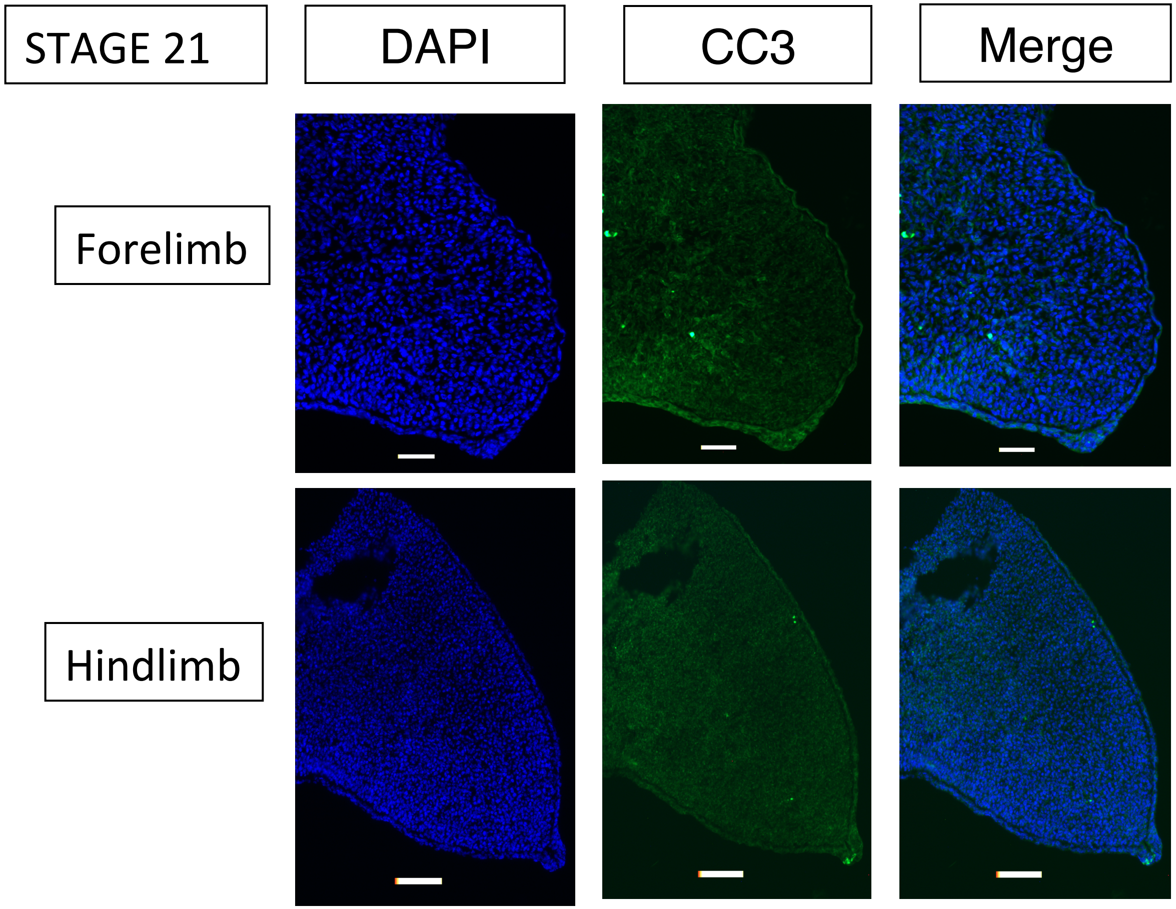

Supplement: Supplementary file 2 — Additional file 2. Staining for apoptosis in embryonic emu limb buds. Immunofluorescent detection of Cleaved Caspase 3 (CC3). Very few apoptotic cells are detected in forelimb and hindlimb buds. Data for stage 21 is shown. Results were the same for other stages. [file 13227_2016_63_MOESM2_ESM.pdf]
